# Pediatric Inflammatory Multisystem Syndrome Temporally Associated With SARS-CoV-2 (PIMS-TS) and Serous Effusions in a Child With Severe Hypoalbuminemia: A Case Report

**DOI:** 10.7759/cureus.38440

**Published:** 2023-05-02

**Authors:** Zohair El Haddar, Aziza Elouali, Ilham Belga, Maria Rkain, Abdeladim Babakhouya

**Affiliations:** 1 Pediatrics, Mohammed VI University Hospital, Oujda, MAR; 2 Pediatrics, Faculty of Medicine and Pharmacy, Mohammed Ist University, Oujda, MAR

**Keywords:** kawasaki disease, serous effusion, covid-19, hypoalbuminemia, pims-ts

## Abstract

In April 2020, Pediatric Inflammatory Multisystem Syndrome temporally associated with severe acute respiratory syndrome coronavirus 2 or SARS‑CoV‑2 (PIMS-TS) was described for the first time in children. Since then, many countries have registered hundreds of cases with clinical similarities to Kawasaki disease. We report the case of a five-year-old boy diagnosed with PIMS-TS who presented myocarditis with serous effusions (pleurisy, ascites, pericarditis) due to severe hypoalbuminemia. This case sheds light on the importance of hypoalbuminemia in evaluating the severity of PIMS-TS and preventing its complications. The patient was successfully treated with intravenous immunoglobulins and oral prednisone.

## Introduction

In April 2020, a new pediatric syndrome related to coronavirus disease 2019 (COVID-19) was described in children and named Pediatric Inflammatory Multisystem Syndrome temporally associated with severe acute respiratory syndrome coronavirus 2 or SARS‑CoV‑2 (PIMS-TS) [1]. The clinical signs of PIMS-TS are almost the same as Kawasaki disease, hence the name Kawasaki-like disease. This includes cutaneous rash, cheilitis, conjunctivitis, prolonged fever, lymphadenopathy, and sometimes gastrointestinal signs and cardiac shock [2]. The biological features of PIMS-TS are important to know to objectively assess its progression and the risk of possible complications. Here, we describe a case of a five-year-old male presenting signs of PIMS-TS with serous effusions due to severe hypoalbuminemia, which was successfully treated with intravenous immunoglobulins and oral prednisone.

## Case presentation

**Figure 1:**
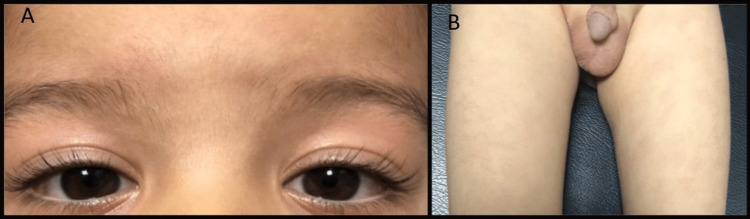
Clinical signs in our case, A: bilateral conjunctivitis, B: hydrocele + maculopapular lesions

A five-year-old Moroccan child, with no known family history of genetic or systemic disease, presented to the pediatric emergency department with a prolonged fever lasting for 15 days that was resistant to paracetamol and antibiotics. The fever was associated with skin rash, vomiting, and deterioration in the general condition. Notably, a history of exposure to respiratory viral infection COVID-19, 20 days before this presentation was mentioned by the family. On physical examination, the patient was asthenic, pale, febrile at 39,6°C, normocardic with a heart rate of 110 beats/minute, normocapnic with a respiratory rate of 25 breaths/minute, and normal oxygen saturation of 98% and good blood pressure. Furthermore, bilateral non-suppurative conjunctivitis was identified, cheilitis, nonpitting edema of the lower limbs, and bilateral symmetric erythematous maculopapular lesions in lower extremities were also noted (Figure 1). Moreover, the abdominal examination revealed hepatomegaly, with diffuse abdominal dullness.

**Figure 2:**
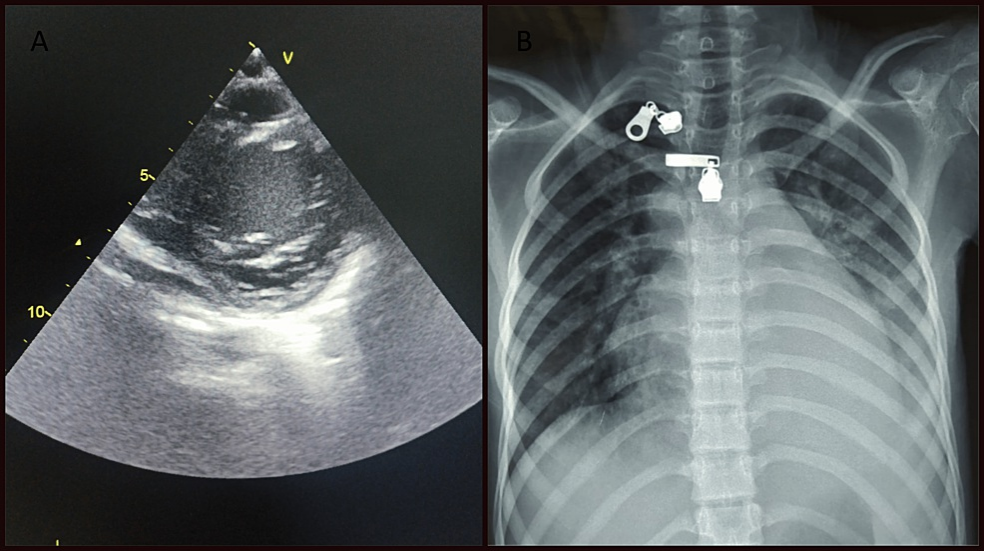
Radiological signs in our case, A: pericarditis on echocardiography; B: pleurisy on chest X-ray

Biologically, hypochromic microcytic anemia with a hemoglobin level of 9 g/dl, thrombocytopenia at 67G/ml, an elevated white blood cell count of 15.5 G/l, and a high level of C-reactive protein (CRP) at 164 mg/L, as well as a high erythrocyte sedimentation rate (ESR) at 85 mm/h, were found. Furthermore, hypoalbuminemia at 19 g/l and positive SARS-CoV-2 serology (immunoglobulin G (IgG) enzyme-linked immunosorbent assay ELISA) with an increased level of pro-B-type natriuretic peptide (Pro-BNP) at 9791 pg/ml were also found. Procalcitonin and fibrinogen levels were negative, and renal and hepatic function tests showed no abnormalities. The transthoracic echocardiography revealed a dilated left ventricle, a low left ventricular ejection fraction at 30%, myocarditis, pericarditis, mitral regurgitation, 3.5 mm right coronary artery dilatation, and an echography objectified hydrocholecystis, a mild peritoneal effusion, and a pleural effusion of medium abundance on the thoracic radiography (Figure 2).

At this stage, the patient was highly suspected of having multisystem inflammatory syndrome in children associated with COVID-19 (MIS-C). Therefore, a therapeutic protocol based on intravenous immunoglobulin (IVIg) at a dose of 2 g/kg, associated with oral acetylsalicylic acid 50 mg/kg/day during the acute phase, and prednisone 2 mg/kg/day orally for seven days was started on the second day after admission. After five days, the dose of acetylsalicylic acid was decreased to 5 mg/kg/day (anti-aggregating dose).

**Figure 3:**
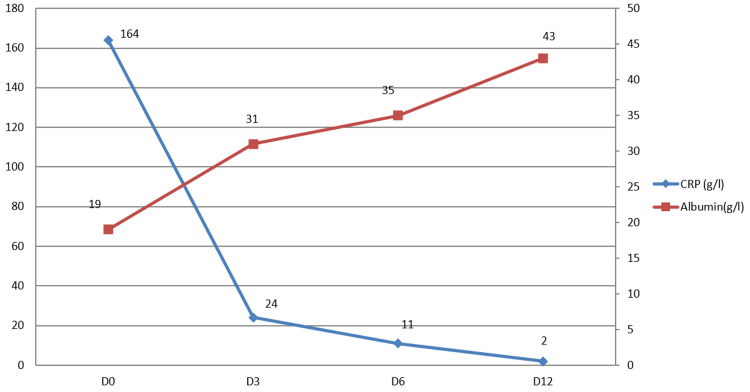
The evolution of CRP and albumin levels in our case CRP: C-reactive protein

During follow-up, a noticeable clinical and biological improvement was observed. The patient became afebrile and non-asthenic, with total regression of the eruption, ascites, and other effusions. The CRP and other inflammatory biomarkers decreased while albuminemia increased to reach its normal value (Figure 3). At the three-month follow-up visit, the patient remained asymptomatic and the echocardiography showed no abnormalities.

## Discussion

Since its identification in December 2019, SARS-CoV-2 has never stopped surprising the world with its different aspects and complications. The newest and most severe complication so far is PIMS-TS, with an incidence of 2-5% [3]. First described in April 2020 in the United Kingdom (UK) and Italy, the etiopathogenesis of this condition is still not yet proven, but some genetic factors are supposed to be responsible for innate immunity alter the immune response to SARS-CoV-2 [4].

PIMS is characterized by hyperinflammation with multiorgan damage. It occurs with persistent fever for three days in a child or adolescent aged less than 21 years along with two of the following signs [5]: cutaneous signs, such as rash, bilateral aseptic conjunctivitis, or mucocutaneous inflammation, hypotension, or shock; and biological or echocardiographic signs of myocardial dysfunction, pericarditis, valvulitis, or coronary abnormalities, evidence of coagulopathy, and acute gastrointestinal symptoms (diarrhea, vomiting, or abdominal pain). Moreover, these signs are associated with biological signs, including elevated markers of inflammation such as erythrocyte sedimentation rate (ESR), C-reactive protein, or procalcitonin, absence of microbial cause of inflammation, including bacterial sepsis, staphylococcal, or streptococcal shock syndromes, evidence of SARS-CoV-2 (reverse transcription-polymerase chain reaction (RT-PCR), antigen test, or serology positive), or likely contact with patients with COVID-19.

Sometimes, it’s called Kawasaki-like disease because of the large similarity with Kawasaki disease. However, the few differences between these two syndromes are mainly the older age in PIMS-TS compared to Kawasaki disease. Gastrointestinal and neurological signs are more frequent in PIMS-TS. Additionally, myocarditis is more common and severe in PIMS-TS. Hypoalbuminemia, lymphopenia, and renal insufficiency are also more apparent [6,7]. This clinical case sheds light on hypoalbuminemia as an important feature in PIMS-TS, associated in most cases with the severe evolution of this syndrome [7], which fits with the results of the literature [6,7].

Clinically, the patient met the criteria of PIMS-TS, including the cutaneous, gastrointestinal, and biological signs. In addition, serous effusions (pleural, pericardial, and peritoneal) were the main characteristics of this case, explained by severe hypoalbuminemia. Mechanisms such as a capillary leak, decreased liver synthesis, increased scavenging, and degradation of albumin are established in the pathophysiology of hypoalbuminemia in inflammatory conditions.

Albumin is the most abundant protein in the plasma, which plays a crucial role in maintaining the oncotic pressure in vessels. In cases of systemic inflammation, the dynamic between the intravascular and extravascular compartments becomes altered, leading to increased capillary leakage of albumin into the extravascular spaces, resulting in reduced serum albumin concentration [8].

Hypoalbuminemia indicates the severity and prognosis of the underlying disease in both adults and children. It is considered a predictor of adverse outcomes and a significant marker of morbidity and mortality, especially in COVID‐19 patients, which may predispose to a poor prognosis [9,10].

## Conclusions

PIMS-TS can be fatal in children due to its various complications. Knowing the features of this syndrome has become necessary as the world continues to record daily new COVID-19 cases. Clinicians should consider hypoalbuminemia as a biomarker of the severity of PIMS-TS, in addition to other cardiac and inflammatory parameters, for better management of this new syndrome.
